# Differential Responses of Methionine Sulfoxide Reductases A and B to Anoxia and Oxidative Stress in the Freshwater Turtle *Trachemys scripta*

**DOI:** 10.3390/metabo11070458

**Published:** 2021-07-16

**Authors:** Melissa Reiterer, Lynsey Bruce, Sarah Milton

**Affiliations:** Department of Biological Sciences, Florida Atlantic University, Boca Raton, FL 33431, USA; mreitere@fau.edu (M.R.); lynseyebruce@gmail.com (L.B.)

**Keywords:** anoxia, antioxidant, epigallocatechin gallate, EGCG, FOXO3a, MsrA, MsrB

## Abstract

Oxidative stress has been acknowledged as a major factor in aging, senescence and neurodegenerative conditions. Mammalian models are susceptible to these stresses following the restoration of oxygen after anoxia; however, some organisms including the freshwater turtle *Trachemys scripta* can withstand repeated anoxia and reoxygenation without apparent pathology. *T. scripta* thus provides us with an alternate vertebrate model to investigate physiological mechanisms of neuroprotection. The objective of this study was to investigate the antioxidant methionine sulfoxide reductase system (Msr) in turtle neuronal tissue. We examined brain transcript and protein levels of MsrA and MsrB and examined the potential for the transcription factor FOXO3a to regulate the oxygen-responsive changes in Msr in vitro. We found that Msr mRNA and protein levels are differentially upregulated during anoxia and reoxygenation, and when cells were exposed to chemical oxidative stress. However, while MsrA and MsrB3 levels increased when cell cultures were exposed to chemical oxidative stress, this induction was not enhanced by treatment with epigallocatechin gallate (EGCG), which has previously been shown to enhance FOXO3a levels in the turtle. These results suggest that FOXO3a and Msr protect the cells from oxidative stress through different molecular pathways, and that both the Msr pathway and EGCG may be therapeutic targets to treat diseases related to oxidative damage.

## 1. Introduction

Survival under physiologically stressful conditions such as ischemia, acidosis or hypoxia requires an ability to maintain cellular homeostasis, preventing or ameliorating the activation of cell death pathways while simultaneously upregulating pro-survival mechanisms. Several vertebrate species tolerate not just hypoxia but hours, days and even weeks of complete anoxia; among the best studied are the crucian carp (*Carassius carassius*) and several species of freshwater turtles, including the western painted turtle *Chrysemys picta* and the red-eared slider *Trachemys scripta* [1]. Both the fish and turtle species have evolved this tolerance in order to withstand long northern winters in frozen-over lakes and ponds, where the ice covering creates an extended period of low oxygen without access to the surface. A critical element for this extended anoxic survival is the ability to prevent cell death in the brain, as in mammals, the brain is considered to be the most anoxia-sensitive of all the organs, with a high oxygen demand and low buffering capacity [1]. Survival mechanisms of the turtle have been under investigation for nearly 40 years [2], with the past decade characterized by the increasing elucidation of cellular and subcellular mechanisms that permit cell survival without oxygen.

These processes are aimed not only at promoting cell survival during anoxia itself but also upon reoxygenation. After an anoxic event, reoxygenation occurs within 10 min in the turtle [3]. This suggests the potential for the rapid release of reactive oxygen species (ROS) that could overwhelm antioxidant defenses and result in cell damage or death. The turtle, however, is able to survive bouts of anoxia and reoxygenation without any signs of functional or cellular impairment [4,5]. Previous studies have demonstrated that ROS levels decrease to near zero by 4 h of anoxia in the turtle brain, and reoxygenation returns these only to normoxic levels [6]. The same pattern is observed in primary neuronal cell cultures [6], which suggests that the turtle can prevent ROS overproduction after reoxygenation, and/or that it has high innate defense mechanisms that can prevent or repair ROS damage [6]. Both of these have been shown to occur in the turtle brain [5,7,8], but the mechanisms are not fully elucidated and may be linked to the same protective mechanisms that allow them to survive anoxia, including upregulation of protective MAPK pathways [9,10] and increased HSP expression [11], in addition to the high innate levels of antioxidants.

Along with exogenous or endogenous compounds that directly act to lower ROS levels, the methionine sulfoxide reductase system (Msr) functions to prevent or reverse protein modifications due to ROS oxidation [12]. The amino acid methionine (Met) is highly susceptible to oxidation and this reaction results in the formation of a diastereomeric mixture of methionine-S-sulfoxide (Met-*S*-(O)) and methionine-R-sulfoxide (Met-*R*-(O)) [13]. The Msr system reduces Met-O back to Met by two distinct enzyme families: methionine-S-sulfoxide reductase A (MsrA) reduces Met-*S*-(O), while methionine-R-sulfoxide reductase B (MsrB) reduces Met-*R*-(O) [13,14,15,16,17]. The cyclic oxidation/reduction of Met residues protects cells by (1) the repair of important proteins, since the oxidation of Met residues can alter protein function and may be lethal for the cell unless a mechanism to repair them exists [18]; and (2) keeping critical proteins in their reduced form [19]. Of the two enzyme forms, MsrA has been studied the most extensively, while MsrB was discovered more recently [13] and is less studied in animal systems [20,21,22].

MsrA is found in a variety of tissues including liver, kidney and brain tissues [23] and, in mammals, has been found in both the mitochondria and the cytosol [24,25]. Previous studies suggested that MsrA has a critical role in protection against oxidative stress [23], as the overexpression of MsrA can increase the lifespan or resistance to oxidative stress in numerous model systems including transgenic flies [26], yeast [27], mice [23], rat cardiac myocytes [28], T-lymphocytes [29], PC12 cells [30], lens cells [31] and WI-38 SV40 fibroblast cells [32]. The knockdown of this gene has been observed to render lens cells more sensitive to oxidative stress [31], and its null mutation renders *E. coli* more sensitive to growth inhibition by H_2_O_2_ [33]. MsrA has also been found to play a role in protection against cardiac ischemia/reperfusion. Cultured cardiac myocytes subjected to ischemia/reoxygenation (I/R) showed protection against injury when overexpressing MsrA [28], while cardiac myocytes from MsrA knockout mice could not tolerate physical or oxidative stress [34].

Studies have also characterized MsrB in various organisms including bacteria [35,36], yeast [37], mammals [38], and fruit flies [39]. In mammalian models, MsrB has been found in three isoforms: MsrB1, MsrB2 and MsrB3; MsrB3 has two variations: MsrB3A and MsrB3B [40]. Each isoform is found in different subcellular locations and therefore may have different functions. MsrB1 has been found to target the cytosol and the nucleus, MsrB2 and MsrB3B the mitochondria and MsrB3A the endoplasmic reticulum [13]; however, the potential role of the MsrBs in protection against oxidative stress and aging is not yet clear. Deletion or overexpression of MsrB alone in yeast cells had little impact on the lifespan, though MsrB deletion additively decreased the lifespan in an *msrA−*/*msrB−* double knockout mutant [41]; similarly, studies overexpressing mouse MsrB1, MsrB2 or *Drosophila* MsrB in fruit flies did not have an effect on the lifespan of the organisms [42,43]. On the other hand, overexpression of the human MsrB3A isoform in fruit flies extended their lifespan and delayed the decline in locomotor activity and fecundity in older flies [21], while the combination of caloric restriction and MsrB overexpression synergistically increased the lifespan in yeast [41]. Overexpression of MsrB2 was also found to promote cell survival against H_2_O_2_-mediated cell death and prevented mitochondrial membrane depolarization in the T-lymphocytic leukemia MOLT-4 cell line [44].

The molecular mechanisms of Msr regulation are also still under investigation; one suggested regulatory mechanism of MsrA is the transcription factor forkhead box O (FOXO), with FOXO3 mRNA highly expressed in the brain [45]. FOXO proteins are involved in a wide array of cellular functions including metabolism, proliferation, differentiation, autophagy, apoptosis, cell cycle, DNA damage/repair and lifespan regulation, as well as oxidative stress protection [46,47,48]. The antioxidant role of FOXO is one of its most crucial functions [49], through activation of major antioxidant enzymes such as MnSOD [50,51] and catalase [52,53,54]. In *T. scripta*, FOXO1 is anoxia-responsive in the liver, and FOXO3 was activated in the heart, kidney and liver in response to anoxia, with both increased nuclear levels and higher DNA binding activity [55]. This higher activity was reflected in enhanced levels of FOXO target genes in the liver including the cell cycle inhibitor p27(kip1) gene and *catalase* [55]. Studies in animal models suggested MsrA as another possible target gene of FOXO [56,57]. A study in *C. elegans* showed that daf-16, a homolog to the transcription factor FOXO3a, enhances MsrA transcription during conditions of oxidative stress [56]. In fruit flies, overexpression of the *Drosophila foxo* increases the expression levels of MsrA compared to controls [57], while null mutants have decreased expression. In the turtle, we recently showed that *foxo3a* transcript levels increase slightly but significantly in anoxia (1.4-fold over control), with a further increase to 2.7-fold upon reoxygenation; chemical oxidative stress induced an approximate 11-fold increase above basal, suggesting FOXO to be an important defense against oxidative stress [58]. Overexpression of human *foxo3a* in turtle brain cultures, and the pharmacological induction of native *foxo3a* via treatment with the green tea polyphenol epigallocatechin-3-gallate (EGCG) both greatly reduce cell death in the face of chemical oxidative stress as well [58], and in *Caenorhabditis elegans*, both the mean and maximum lifespan were increased when cultures were grown with EGCG, which increased nuclear FOXO [59]. Two putative FOXO transcription binding sites have been identified in the *T. scripta* MsrA promoter region (sequence kindly provided by Dr. Paul Kirchman), further suggesting that MsrA may be a possible target for FOXO regulation in this organism.

On the other hand, in other animal models, FOXO/DAF-16 is negatively regulated by PI3K-AKT [60] and upregulated by JNK [61,62]. The phosphorylation of FOXO by AKT prevents its translocation to the nucleus and thus inhibits its activity (reviewed in [63]). In *T. scripta* though, AKT levels during anoxia are upregulated, while JNK is downregulated, both in vivo and in vitro [9,10], and blocking pAKT pharmacologically results in increased cell death, while blocking JNK decreases cell death [64]. In addition, earlier studies showed p-FOXO increases in the cytoplasmic fraction of the anoxic *T*. *scripta* brain in vivo in anoxia and after 4 h of anoxia/4 h of reoxygenation (Figure S1), suggesting inhibition of the transcription factor. Together, these would suggest that FOXO would not be the regulatory mechanism behind any changes in Msr. The purpose of this study was thus to determine if Msr levels are upregulated in *T. scripta* undergoing anoxia/reoxygenation, as freshwater turtles have high levels of other antioxidants. We examined both MsrA and MsrB transcript and protein levels in vivo and examined the potential for FOXO to be a regulator of Msr changes in vitro. While mammals exhibit three different isoforms of MsrB [17], other organisms have only one form, including yeast, fruit flies and nematodes [13]. As it is unknown what form(s) and localization of MsrB may be present in the turtle, results are presented for putative MsrB2 levels (based on primers from the freshwater turtle *Chrysemys picta* and binding of mammalian antibodies) as well as a putative MsrB3.

## 2. Results

**Figure 1:**
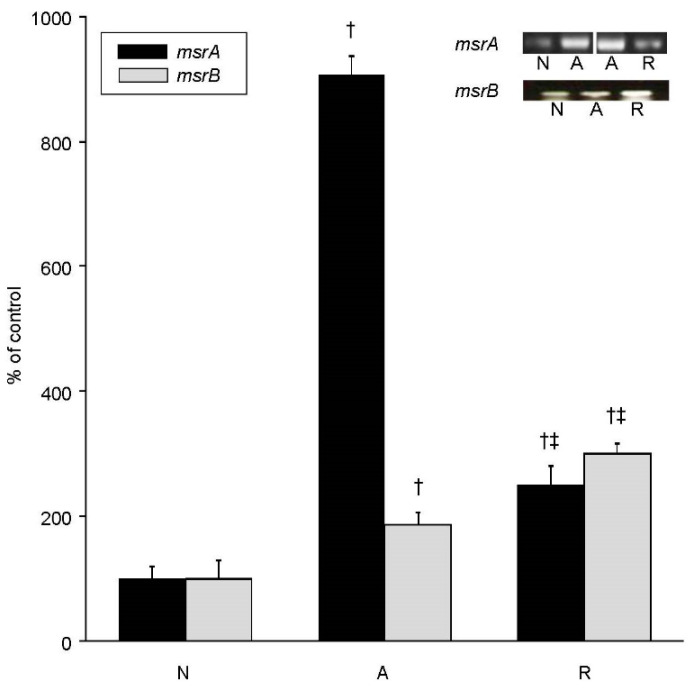
In vivo changes in *msrA* and *msrB2* transcript levels in the whole brain of *T. scripta*. Representative gels of semi-quantitative RT-PCR for *msrA* and *msrB2* and densitometric analysis of mRNA levels expressed as percent of normoxic control. N = normoxic controls, A = 4 h of anoxia, R = 4 h of anoxia/4 h of reoxygenation. Statistics: † = significantly different from normoxic controls, ‡ = significantly different from anoxia (*p* < 0.001, *n* = 3–5 individuals/treatment).

**Figure 2:**
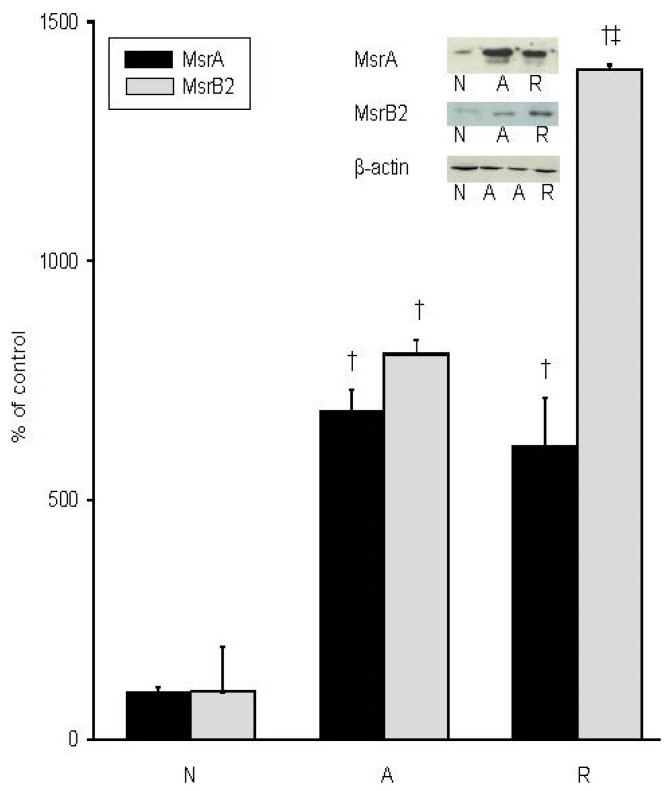
In vivo changes in Msr protein levels in the whole brain of *T. scripta*. Representative immunoblots of whole brain MsrA and MsrB2 proteins and actin control, and densitometric analysis of changes. N = normoxic controls, A = 4 h of anoxia, R = 4 h of anoxia/4 h of reoxygenation. Statistics: † = significantly different from normoxia; ‡ = significantly different from anoxia (*p* < 0.001, *n* = 5 individuals/treatment).

We found that *mrsA* and *msrB2* transcript levels are differentially upregulated during anoxia and reoxygenation in the whole brain of the freshwater turtle *Trachemys scripta*, with *msrA* levels increasing 9-fold during anoxia, then decreasing during reoxygenation to only 2.5-fold higher than normoxia (Figure 1). The expression of *msrB2* increased 2-fold during anoxia and increased further to 3-fold of normoxic controls during reoxygenation. MsrA whole brain protein levels reflect the trend evident in the mRNA transcripts during anoxia, with immunoblotting results suggesting MsrA increased nearly 7-fold over basal by 4 h of anoxia (Figure 2 and Figure S2). However, despite the decrease in mRNA upon reoxygenation, the MsrA protein levels remained near anoxic levels, at 6-fold over basal (Figure 2). Changes in putative MsrB2 protein levels were even more dramatic. By 4 h of anoxia, MsrB2 levels were nearly 8-fold higher than controls, with a further elevation to nearly 15-fold of basal upon reoxygenation (Figure 2 and Figure S3). Actin levels remained unchanged throughout each condition [65], suggesting that the change in Msr protein levels was specific and not due to a generalized protein response to anoxia/reoxygenation (Figure 2 and Figure S4).

Even though the significant increases in Msr levels observed suggest a neuroprotective role for both MsrA and MsrB during anoxia and/or reoxygenation, the mechanism by which these increases occur is still unknown. Studies in *C. elegans* have shown that daf-16, a homolog to the transcription factor FOXO3a, enhances MsrA transcription during conditions of oxidative stress, and in results already published, we showed that *foxo3a* transcript levels increase in vitro in anoxia and under conditions of oxidative stress [57] in primary cell cultures derived from whole turtle brains. As this increase protects the cells against severe oxidative stress, we thus looked to see if the same drivers of *foxo* expression would also affect Msr levels in whole brain primary cultures.

**Figure 3:**
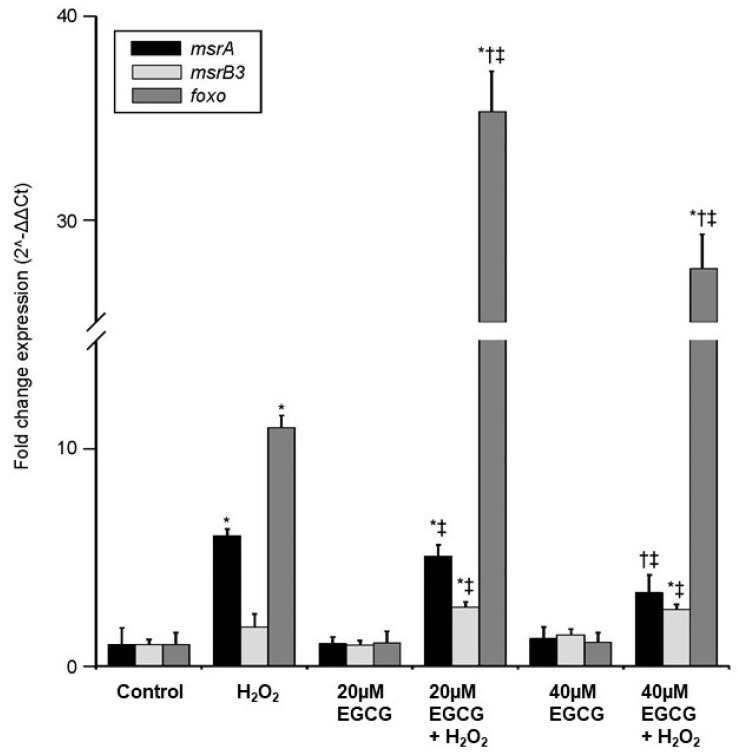
EGCG treatment does not enhance the upregulation of *msrA* or *msrB3* under oxidative stress conditions in whole brain cell cultures. *MsrA* and *foxo3a*, but not *msrB3*, levels are upregulated by H_2_O_2_ treatment, but EGCG treatment alone does not significantly affect *msrA*, *msrB3* or *foxo3a* expression. While pre-treatment with EGCG enhances the expression of *foxo3a* beyond stimulation with H_2_O_2_ alone, *msrA* expression in cultures treated with 20 µM and H_2_O_2_ is no different than H_2_O_2_ alone, while 40 µM EGCG plus H_2_O_2_ is slightly but significantly lower. *msrB3* expression in cultures treated with either 20 µM or 40 µM EGCG and H_2_O_2_ is no different than H_2_O_2_ alone. * = significantly different from control, † = significantly different from H_2_O_2_ treatment, ‡ = significantly different from same EGCG treatment without H_2_O_2_. (*p* < 0.05). Foxo data modified from [58].

We demonstrated earlier that, while anoxia alone does not increase it, *foxo3a* expression in cultures made from the whole brain is upregulated 11-fold by exposure to H_2_O_2_, with even more significant increases when treated with EGCG in addition to H_2_O_2_ [58]. Extending that study, we found that the extensive upregulation of *foxo3a* in response to EGCG + H_2_O_2_ is not reflected in changes to *msrA* or *msrB3* transcript levels as detected by quantitative PCR (Figure 3). While *msrA* levels are upregulated by the addition of H_2_O_2_ alone (6.0 ± 0.3-fold increase from basal), there was no additional increases resulting from EGCG exposure. Upregulation of *msrA* is observed only when cell cultures pre-treated with EGCG are also exposed to oxidative stress, and this upregulation is not significant compared to H_2_O_2_ treatment alone (20 µM EGCG 5.1 ± 0.5-fold increase from basal), and is actually slightly but significantly lower in cell treated with 40 µM EGCG plus H_2_O_2_ (3.4 ± 0.8-fold increase from basal) (Figure 3). Similarly, *msrB3* levels are significantly upregulated from basal only when cells pre-treated with EGCG are also subjected to H_2_O_2_; however, this upregulation is also not significantly different from treatment with H_2_O_2_ alone (H_2_O_2_ 1.8 ± 0.6-fold increase from basal, 20 µM 2.7 ± 0.3-fold increase from basal and 40 µM 2.6 ± 0.3-fold increase from basal) (Figure 3). Thus, the significant increases in *foxo3a* expression resulting from EGCG and oxidative stress are not reflected in similar increases in Msr expression, suggesting that these proteins are not downstream targets of FOXO in the turtle.

## 3. Discussion

Here, we provide evidence of oxygen-regulated changes in MsrA and MsrB transcript and protein levels in the turtle brain. While anoxia induced an upregulation of both MsrA and MsrB, reoxygenation resulted in decreased *msrA* transcript levels, but a continued upregulation of *msrB*, with protein levels for both remaining elevated above normoxic controls. While the general suppression of both transcription and protein synthesis is a key mechanism utilized to decrease the energy demand among invertebrate and vertebrate hypoxia-tolerant organisms [66,67,68,69], it is apparent from this and other studies that critical proteins in the turtle are upregulated against the general background of suppression in anoxia [9,65,70,71]. Such upregulation permits survival in the face of anoxia and potential ROS stress, and it has been suggested that high antioxidant levels, coupled to the ability to suppress ROS formation upon reoxygenation [6,72], may be an important part of turtle longevity as well [73]. The significant upregulation of both Msrs may contribute to this protection, as induction of MsrA has been shown to increase the lifespan in other animal models [41,56]. As it has been suggested in a variety of other organisms, the upregulation of such antioxidants during anoxia also supports the hypothesis that turtles exhibit the preparation for oxidative stress (POS) response, where adapted organisms prevent the accumulation of toxic ROS upon reoxygenation by increasing antioxidant defenses during the environmental stress event [74]. While the apparent protein level increases in MsrA and MsrB2 are both substantially greater than transcript levels, immunoblotting using mammalian antibodies in the turtle may not allow precise quantification of results; the increases in transcript levels may have resulted in more modestly elevated protein levels than reported here. Thus, while we are confident of the trends presented, it would be of interest for future work to measure specific enzyme activities of both MsrA and MsrB.

While we are currently investigating the function of the Msr proteins in the turtle brain, the differential changes suggest individual roles for MsrA and MsrB, with the substantial increase in MsrB under reoxygenation, in particular, suggesting a critical neuroprotective role during times of potential oxidative stress. Others investigating the role of MsrB as a protective mechanism against oxidative stress or aging in different model organisms reported mixed findings. Silencing any of the three mammalian forms of MsrB (-B1, -B2 or -B3) in human lens cells increased their sensitivity to oxidative stress-induced cell death [75], but in the fruit fly *Drosophila melanogaster*, some investigators reported that MsrB protects against oxidative stress and aging [21], and others reported that overexpression had no effect on the lifespan [43]. Bruce et al. [76], on the other hand, found that the lifespan was only affected when both *msrA* and *msrB* were deleted in *D*. *melanogaster*.

While the protective effect of the Msr family has been well described in multiple organisms from bacteria to mice [26,31,34,41,77], the mechanism which regulates Msr transcription and translation has been far less studied. The only other vertebrate thus far to also show an oxygen-responsive regulation of Msr during hypoxia is the subterranean mole rat *Spalax galili* [78], which is also tolerant to severe hypoxia [79,80]. In other organisms, hypoxia inducible factor HIF-1α has been suggested as a possible MsrA regulator due to its role in the upregulation of multiple protective genes during hypoxia [81,82]. However, HIF-1α transcript levels do not increase in the anoxic turtle [83], nor do protein levels even over 24 h of anoxia or upon reoxygenation [84]. Earlier results from our lab, in fact, showed that HIF-1α disappears from the nuclear fraction of neuronal cultures in anoxia (Milton, unpublished data), and rather than being protective, stimulation of HIF-1α expression pharmacologically with tilorone increased cell death in a dose-dependent manner, concomitant with an increase in anoxic HIF-1α levels in the nucleus [84]. Studies in similarly anoxia-tolerant crucian carp have shown that HIF-1 DNA binding activity in severely hypoxic warm-acclimated (26 °C) fish also remained unaltered in all tissues studied [85]. As many of the pro-survival pathways upregulated by HIF-1 in hypoxia in mammalian models are conversely downregulated in good facultative anaerobes as a means to conserve energy in a deeply depressed metabolic state [1], the lack of a strong HIF-1 response in anoxia in these models is unsurprising.

As the lack of an increase in HIF-1α when Msr levels are changing in both anoxia and reoxygenation suggests, therefore, that this transcription factor is unlikely to be the regulator of oxygen-modulated Msr expression, we investigated whether Msr levels might, instead, be affected by the transcription factor FOXO3a, as its homolog daf-16 is involved in the regulation of MsrA expression in *C. elegans* [56]. In addition, knockdown of FOXO decreased both MsrA and MsrB mRNA levels and abolished the induction by paraquat of both Msrs in the red flour beetle [86]. An analysis of upstream regulatory sequences of the turtle *msrA* gene also suggested the presence of candidate FOXO binding sites (Kirchman, pers. comm.).

FOXO3a has also been demonstrated to render protection through the upregulation of other antioxidant enzymes such as Mn SOD [50,51,87] and CAT [52,53,88] during conditions of oxidative stress, and in mammalian models, oxidative stress or heat shock results in the movement of FoxO3a from the cytoplasm to the nucleus [89,90]. The *catalase* gene also appears to be one target of anoxia-induced upregulation of FOXO3 in the nucleus of turtle kidney, heart and liver cells [55]. In addition, *foxo3a* levels in turtle brain cultures are significantly upregulated during reoxygenation and are protective against chemical oxidative stress [58]. Based on these studies and on the putative binding sites of FOXO3a on the MsrA promoter, FOXO3a appeared to be a good candidate for the regulator of Msr expression, but the results of this study suggest otherwise. In this study, however, we found that pharmacological manipulation of FOXO3a with EGCG increased its expression during oxidative stress conditions but did not result in an increased expression of either MsrA or MsrB. The data thus imply that FOXO3a and Msr operate by separate molecular pathways in this model, at least in brain tissue. As it was suggested by Krivoruchko and Storey [55] in other turtle organs in anoxia, FOXO3a may be protecting the cells by activating other target genes involved in different cellular processes that have the common end goal of cellular protection and survival [87,88,89,91,92,93].

The mechanisms behind MsrB’s responses to oxidative stress are even less studied than for MsrA. A study in *E. coli* demonstrated that the antibiotic fusaricidin A can activate both recombinant MsrA and MsrB [94], while in fruit flies, it is under hormonal control [95]. In this study, the MsrB protein levels appeared to change more significantly than the MsrB messenger level, suggesting that there is regulation of MsrB activity at the translational level or in the rate of protein degradation. Picot et al. [96] reported decreases in both mitochondrial and cytosolic MsrA activity during ischemia and early reperfusion in the rat heart, though there was no change in Msr levels, which suggests further regulation at the translational level is possible, and in mammalian models, the decrease in MsrA activity would diminish the cellular antioxidant response and reduce recovery. Since it has also been reported that MsrB expression may be controlled by MsrA expression [97], the MsrA increases in the anoxic turtle brain could also have led to the continued elevation in MsrB expression. The significant changes to the Msrs, and the likelihood that they play an important role in resistance to hypoxia, anoxia and/or oxidative stress suggest it would be of interest to further investigate the relationship between Msr and other neuroprotective mechanisms that are activated during anoxia and reoxygenation, as well as determining the underlying regulatory mechanism. While this study focused on FOXO3a, there is, of course, a suite of other transcription factors that could potentially play this role.

These observations further elucidate cell survival pathways in response to hypoxia/ischemia and may lead to novel therapeutics based on the repair of methionine oxidation in key survival pathways. Understanding the neuroprotective mechanisms in the turtle could reveal new possibilities for defense against hypoxia/anoxia and oxidative stress in mammalian models and may help determine future strategies to ameliorate the damaging effects of ROS overproduction, along with the neuropathologies associated with protein oxidation [98]. This vertebrate system, in which Msr is upregulated in response to physiological conditions, provides a unique model in which to further examine the signal transduction pathways that regulate Msr expression. In addition, the protective role of EGCG and its ability to upregulate FOXO suggest it should be further studied as a therapeutic target to treat diseases of oxidative stress.

## 4. Materials and Methods

This study was approved by the Florida Atlantic University Institutional Animal Care and Use Committee as IACUC protocols A15-35 and A18-38, approved 10/2015 and renewed 10/2018.

### 4.1. Animals

Freshwater turtles (*Trachemys scripta*) weighing 300–500 g were obtained from commercial suppliers (Clive Longdon, Tallahassee, FL, USA; Niles Biological, Inc., Sacramento, CA, USA) and kept in freshwater aquaria in an in-lab facility on a 12 h light/dark cycle. Animals were fed three times weekly to satiety with commercial turtle food.

### 4.2. Protein Extraction from Whole Brain

Turtles were individually placed in sealed 2 L plastic chambers at room temperature (24 ± 1 °C). The three experimental sets (*n* = 5) included normoxic controls, anoxic animals exposed to 4 h of 99.99% N_2_ (positive pressure flow-through, Air Gas, Miami, FL, USA) and a third group of 4 h of anoxia/4 h of normoxic recovery in room air. Normoxic control animals were taken directly from aquaria. Animals were then sacrificed by decapitation, and the brains were immediately homogenized for protein extraction. Whole brains were homogenized and extracted with 200 μL lysis buffer (5 mM EDTA, pH 8.0, 0.15 M NaCl, 1% Triton X-100, 10 mM Tris-Cl pH 7.4), as previously described [70]. Protein concentrations were determined using a standard BCA assay following the manufacturer’s protocol (Pierce Biotechnology, Inc., Rockville, IL, USA).

### 4.3. Cell Culture Cultivation and Treatment

For whole brain cell cultures, the cortex of the turtle brain was aseptically chopped and added to a cocktail of MEM, 0.16 U/mL Dispase (Gibco, Waltham, MA, USA), 12.5 U/mL Collagenase (Gibco) and 6.25 mg/mL Hyalurondinase (Sigma-Aldrich, St. Louis, MO, USA). The solution was placed on a rocker for 4 h at 37 °C and resuspended every 15 min for 1 h. The homogenate was then centrifuged at 750× *g* for 6 min, the supernatant was discarded and the pellet was washed with fresh MEM. A second centrifugation at 750× *g* for 6 min was performed, the supernatant was discarded and the pellet was removed and placed into MEM with 10% FBS and 1% Pen/Strep and then plated into 6-well plate culture dishes. The media in each well were changed on the following day and weekly afterwards. Cell cultures were grown for 3 weeks at 30 °C in a 5% CO_2_ incubator. Anoxia and anoxia/reoxygenation in cell cultures were induced in a Shel-Lab Bactron anaerobic chamber (Sheldon Manufacturing Inc. Cornelius, OR, USA) at 30 °C under an anoxic gas mixture (90% N_2_, 5% He, 5% CO_2_, AirGas, Miami, FL, USA), as previously described [10]. An OM-4 oxygen meter (Microelectrodes Inc., Bedford, NH, USA) was used to continuously monitor oxygen levels within the chamber. Control cultures were used directly from the incubator.

### 4.4. Protein Extraction from Culture

Protein was extracted from cell cultures (*n* = 5) using RIPA lysis buffer (0.15 M NaCl; 5 mM EDTA, pH 8.0; 1% Triton X100; 10 mM Tris-Cl, pH 7.4), and 5 M DTT, 100 mM PMSF and 5 M mercaptoethanol at a 1:1000 ratio of the RIPA lysis buffer. Cells were scraped using sterile cell scrapers and allowed to incubate on ice for 10 min before being centrifuged at 18,500× *g* for 10 min at 4 °C, and the supernatant was collected. The protein concentration was determined using a standard BCA Protein Assay (Pierce Biotechnology).

### 4.5. RT-PCR

Total RNA was extracted using TRIzol reagent (Life Technologies, Grand Island, NY, USA) and treated with DNase I. RNA quality was analyzed on a 1% denaturating EtBr-agarose gel, and the quantity was measured using Gene Quant pro (Amersham Pharmacia Biotech, Uppsala, Sweden). For MsrA, cDNA was synthesized from total RNA using a random MsrA forward primer for MsrA, and an actin forward primer for actin. The PCR reactions using Taq polymerase were as follows: MsrA: denaturation for 3 min, 95 °C; PCR: 45 cycles (3 min, 95 °C; 30 s, 59 °C; 1 min, 72 °C), followed by elongation: 10 min, 72 °C. MsrB: denaturation for 7 min, 94 °C; PCR: 40 cycles (1 min, 94 °C; 45 s, 55 °C; 1.0 min, 72 °C), followed by elongation: 10 min, 72 °C. For actin, PCR was conducted for 40 cycles (1 min, 94° C; 2 min, 57 °C; 3 min, 72 °C). The following primers were employed: MsrA: 5′-TTCTGTTGTGATT-GTGCCAAA-3′ (forward) and 5′-GGACACAGAT-GGTTTTATTTGGT-3′ (reverse); actin primers: 5′-CACCAACTGGGACGACATGG-3′ (forward) and 5′-GTCGGC-CAGCTCGTAGCTCT-3′ (reverse). For MsrB, a comparative alignment was performed between species (*E. coli*, mouse, human) where MsrB has been sequenced, searching for conserved regions using ClustalX v.1.81 and Genedoc v.2.6.002 (http://www.psc.edu/biomed/genedoc, accessed on 16 February 2009). Primers specific to turtle MsrB cDNA were designed from a partial cDNA sequence that was obtained previously by RT-PCR analysis of turtle brain mRNA using degenerate primers homologous to MsrB sequences from frog, mouse and human. Turtle brain-specific MsrB primers were: 5′-TCCACGTTAGTCCCTGTTCA-3′ (forward) and 5′-CTTTGAGCGTCTCGAATCT-3′ (reverse). Controls in which RNA or RT was omitted from the RT reaction were carried out to confirm the absence of residual genomic DNA. Following electrophoresis of PCR products, gels were stained with ethidium bromide and digitally photographed for quantification using NIH Image J 1.60 software. For semi-quantitative assessment of MsrA and MsrB RT-PCR, signal intensities were expressed as a ratio of levels of PCR products amplified from turtle actin cDNAs.

### 4.6. Immunoblotting

Equal amounts of protein from cell lysates were separated electrophoretically by SDS-PAGE (12%) at 150 V for 1 h and subsequently transferred to nitrocellulose membranes (Hybond ECL, Amersham Biosciences, Piscataway, NJ, USA). Actin (Sigma-Aldrich; 1:3000) was used as a control in all instances to verify equal loading of proteins. Membranes were blocked in 5% nonfat dried milk in Tris-buffered saline (25 mM Tris-Cl, pH 7.5, at 24 °C, 150 mM NaCl) with 0.1% Tween 20 for 1 h at room temperature. Primary antibodies were diluted in 5% milk and incubated overnight at 4 °C. The primary antibodies used included: methionine sulfoxide reductase A (Abcam, Cambridge, MA, USA; 1:1000), MsrB2 (Epitomics, Burlingame, CA, USA, 1:1000) and β-actin (Abcam, 1:3000). Secondary antibodies (goat anti-rabbit and rabbit anti-mouse) were obtained from Southern Biotech (Birmingham, AL, USA). The membranes were washed 3× with TBST and incubated for 1 h with an HRP-conjugated secondary antibody (Southern Biotech; Millipore, Billerica, MA, USA) diluted in 5% milk. The protein–antibody complexes were visualized by ECL chemiluminescence (Amersham Biosciences, Amersham, HP, UK) and quantified by relative densitometry using NIH ImageJ image analysis software. Preliminary runs established the range of protein loading and antibody concentrations to remain in the linear range of the signal (Figure S5). Results were normalized to percent of actin (no change in anoxia, relative changes expressed as percent of control (Figure 2 and Figure S4)).

### 4.7. Chemical Oxidative Stress

As turtle cell cultures are not susceptible to damage by straightforward anoxia/reoxygenation, we utilized 1.4 mM H_2_O_2_ as chemical oxidative stress, as previously described [58]. This resulted in ~75% cell death within 30 min. Cell cultures where then extracted for RNA isolation.

### 4.8. Pharmacological Stimulation of FOXO3a with EGCG

EGCG was purchased from SigmaAldrich (St Louise, MO, USA). Cell cultures were grown to 50–70% confluency and exposed to the following treatments: 1.4 mM H_2_O_2_, 20 μM EGCG, 20 μM EGCG + 1.4 mM H_2_O_2_, 40 μM of EGCG and 40 μM of EGCG + 1.4 mM H_2_O_2_, as previously described [58]. Concentrations of 20 μM and 40 μM of EGCG were used as these concentrations did not appear to have any negative effects on the cultures (no cell death or change in morphology). Incubation was performed in a rocker at room temperature for 4 h (for EGCG) with an additional 30 min for H_2_O_2_ exposure. Cells were then extracted for RNA isolation.

### 4.9. Reverse Transcription and QPCR

RT was performed using iScript™ Reverse Transcription Supermix for RT-qPCR (Bio-Rad, Hercules, CA, USA) following the manufacturer’s protocol. Briefly, 1 μg of RNA was mixed with iScript™ Supermix and water to a volume of 20 μL, followed by 5 min incubation at 25 °C for priming, 20 min at 46 °C for reverse transcription and 1 min at 95 °C for RT inactivation.

QPCR was performed using SsoAdvanced™ Universal SYBR^®^ Green Supermix (Bio-Rad, Hercules, CA, USA). An amount of 100 ng of cDNA template was mixed with 2× SYBR Green, 100 μM of Forward and Reverser Primers and H_2_O. The amplification cycle consisted of 3 min initial denaturation at 95 °C, followed by 45 cycles of 95 °C for 30 s and 60 °C for 1 min. Turtle-specific primers were created using the PrimerQuest Design tool from Integrated DNA Technologies (Skokie, IL, USA). Actin primers were designed based on the *Chrysemys picta* genome; MsrA and MsrB3 primers were designed based on a *T. scripta* sequence kindly provided by Dr. Kirchman. The following primers were used: MsrA: 5′-TGACCCGACACAAGGAATGAGGCAAGG-3′ (forward) and 5′-TGATTGTGCCAAAACCGCTCTCCGT-CA-3′ (reverse); MsrB3: 5′-AAGGTGGTCT TTTCCCAGCA-3′ (forward) and 5′-GGAGTTCCACAGACAACACAT-3′(reverse); FOXO3a: 5′-CTCAGTCCAACCAGGGAAGTTTG-3′ (forward) and 5′-GGTGACTGCTGCTGGTGTTT-3′ (reverse); actin 5′-CACCACAG-CCGAAAGGGAAAT-3′ (forward) and 5′-CATCAGGGAG TTCGTAGCTCTTCT-3′ (reverse). Actin was used as a housekeeping gene as there is no change in its expression during anoxia.

### 4.10. Statistical Analysis

Statistical significance was evaluated using ANOVA followed by Tukey’s post hoc test. A value of *p* < 0.05 was used to denote statistical significance, and results are expressed as mean ± S.E.M. All statistics were carried out using SigmaPlot 11.0 (Systat Software Inc., San Jose, CA, USA) statistical software.

## Data Availability

Data are contained within the article and Supplementary Materials.

## Supplementary Materials

The following are available online at https://www.mdpi.com/article/10.3390/metabo11070458/s1, Figure S1: Immunoblots from 3 individual turtles suggesting some increase in phosphorylated FOXO (p-FOXO) in the cytoplasm (cyto) of the anoxic and anoxic/reoxygenated turtle *T. scripta*, Figure S2: Upper panels: Western blot of MsrA and actin (as loading control) from whole brains of 3 different turtles. Lower panels: Western blot showing MsrA levels from whole brains of 2 turtles, with cells fractionated into cytoplasmic and mitochondrial compartments, Figure S3: Original immunoblot for MsrB2, Figure S4: Original immunoblot of housekeeping protein β-actin in 4–5 individuals under conditions of normoxia (N), 4 h of anoxia (A) and 4 h of anoxia/4 h of reoxygenation (R), Figure S5: Immunoblotting for MsrB2. Preliminary work was investigating if Msr levels varied differently between the cytoplasmic (“C” lanes) and mitochondrial (“M” lanes) compartments in response to anoxia or anoxia/reoxygenation.
