# Supplementary material for: Differential Responses of Methionine Sulfoxide Reductases A and B to Anoxia and Oxidative Stress in the Freshwater Turtle Trachemys scripta
Source: Metabolites. 2021 Jul 16;11(7):458. doi: 10.3390/metabo11070458 (PMC8304764; doi:10.3390/metabo11070458)
Supplement: Supplementary file 1 [file metabolites-11-00458-s001.zip › metabolites-1265449-supplementary.pdf]

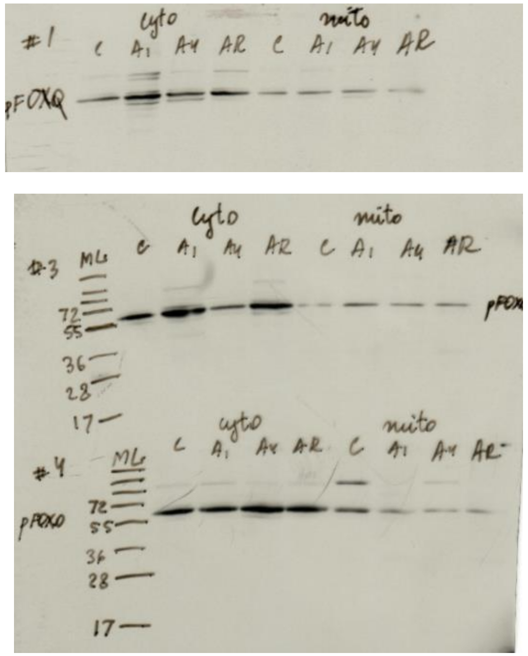

S1: Immunoblots from 3 individual turtles suggesting some increase in phosphorylated FOXO (p-FOXO) in the cytoplasm (cyto) of the anoxic and anoxic/reoxygenated turtle *T. scripta*. Mitochondrial fraction is included for reference (mito). C=control (normoxia), A1 = 1h anoxic exposure A4 = 4h anoxic exposure, AR= 4h anoxia/4h reoxygenation. Phosphorylation of FOXO via the PI3K-AKT pathway results in its nuclear exclusion and thus suppression of FOXO-dependent transcription of target genes.

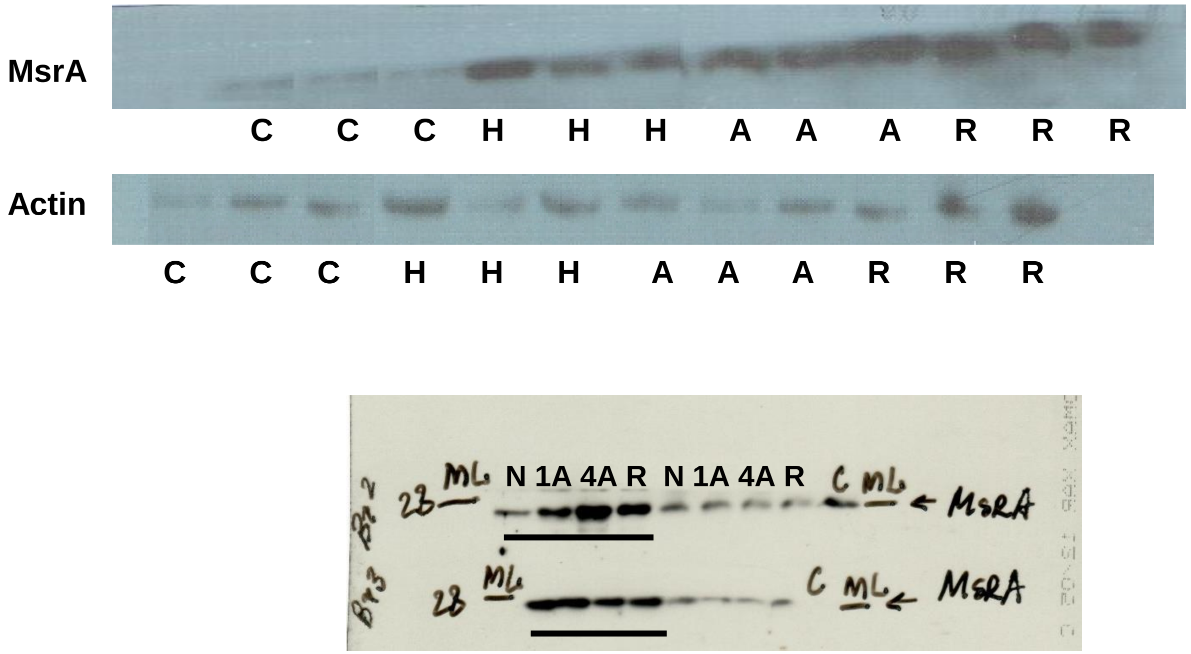

S2: Upper panels: Western blot of MsrA and actin (as loading control) from whole brains of 3 different turtles. Lower panels: Western blot showing MsrA levels from whole brains of 2 turtles, with cells fractionated into cytoplasmic and mitochondrial compartments. Preliminary work was investigating if MsrA levels varied differently between the cytoplasmic (underlined with bar) and mitochondrial (no underline) compartments in response to anoxia or anoxia/reoxygenation. As the MsrA response was present almost entirely in the cytoplasmic fraction, all research reported in this paper was performed on unfractionated cellular homogenates. N=normoxia, 1A = 1h anoxic exposure, 4A = 4h anoxic exposure, R= 4h anoxia/4h reoxygenation.

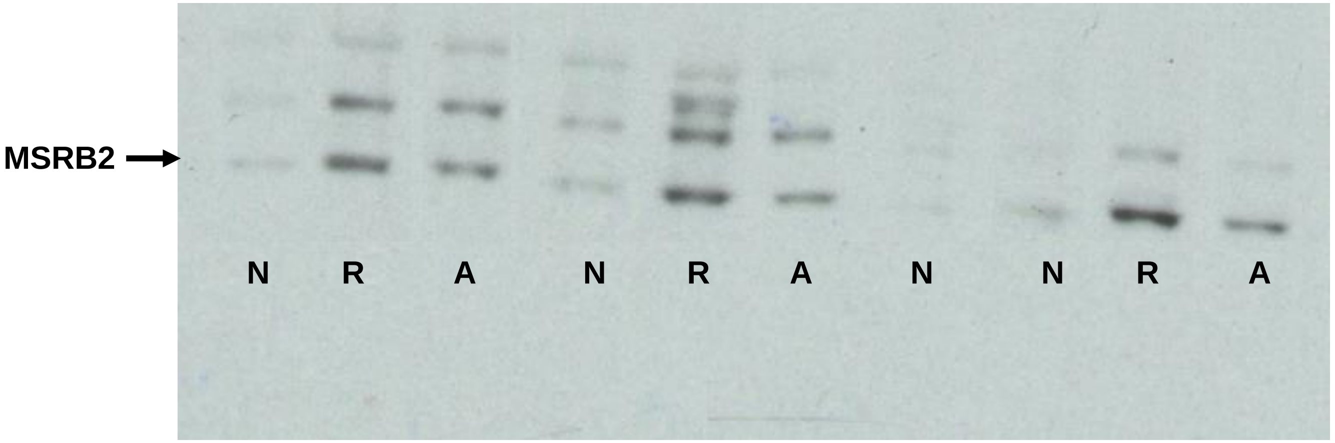

S3: Original immunoblot for MsrB2 (lowest band). N=normoxia, A = 4h anoxic exposure, R= 4h anoxia/4h reoxygenation.

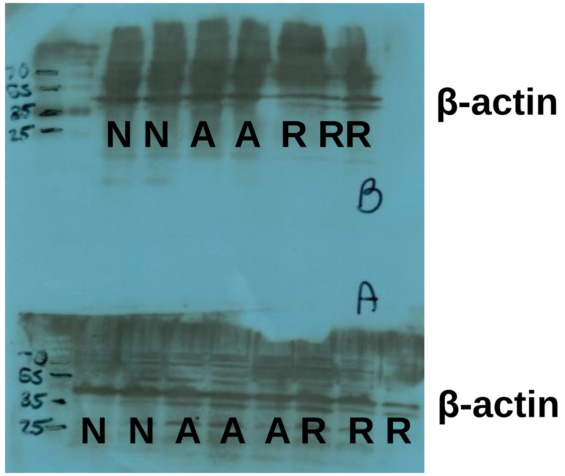

S4. Original immunoblot of housekeeping protein β-actin in 4-5 individuals under conditions of Normoxia (N), 4h Anoxia (A) and 4h Anoxia/4h Reoxygenation (R). Levels of β-actin do not change across these experimental conditions

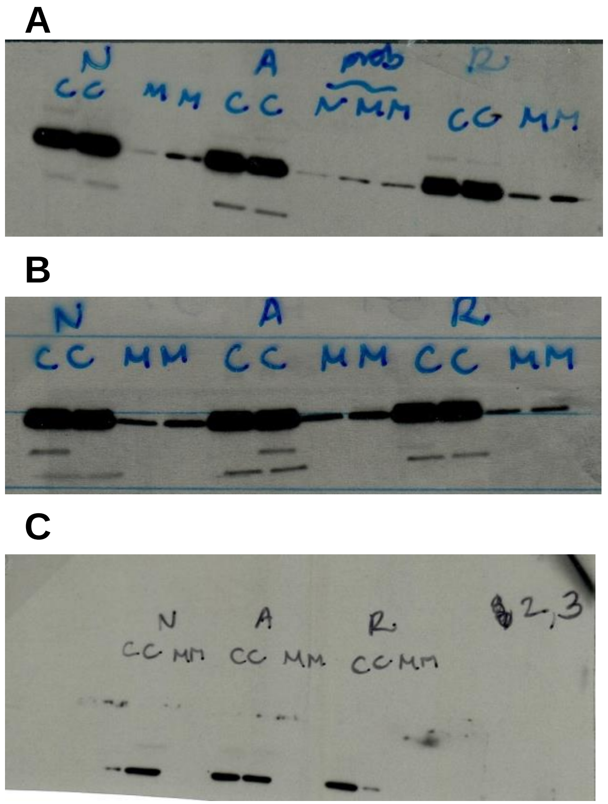

S5. Immunoblotting for MsrB2. Preliminary work was investigating if Msr levels varied differently between the cytoplasmic (“C” lanes) and mitochondrial (“M” lanes) compartments in response to anoxia or anoxia/reoxygenation. Blot A: gels run with 32 ug/total protein/lane. Blot B: gels run with 54 ug/total protein/lane. Blot C: gels run with 10 ug/total protein/lane. As the Msr response was present almost entirely in the cytoplasmic fraction, all research reported in this paper was performed on unfractionated cellular homogenates. N=normoxia, A = 4h anoxic exposure, R= 4h anoxia/4h reoxygenation.
